# Prescribing and Monitoring of Pychotropic Medications in a CAMHS Inpatient Service

**DOI:** 10.1192/bjo.2024.563

**Published:** 2024-08-01

**Authors:** Olorunleke Erunkulu, Shermin Imran, Wasim Ashraf

**Affiliations:** 1 Manchester Foundation NHS Trust, Manchester, United Kingdom; 2 Greater Manchester Mental Health Trust, Manchester, United Kingdom

## Abstract

**Aims:**

To ensure that there is a clear rationale for commencing service users on psychotropic medications.

To ensure that the prescription of psychotropic medications is evidence-based and that they are in line with the Trusts and NICE guidelines.

Ensure that psychotropic medications are regularly reviewed by the managing team.

To ensure that information about medications is adequately shared with patients and carers.

To ensure that service users are well-monitored for side effects.

**Methods:**

A 2-week retrospective audit on Phoenix ward.

Clinical information from all the current service users on psychotropic medication was reviewed.

The clinical information was collated from all 8 service users’ medication cards, ward round documents, MDT reviews, and electronic notes (PARIS), and these were analyzed by the inpatient specialty registrar.

**Results:**

1. We attained a 100% mark in some areas of our prescribing such as indicating the rationale, the maximum dose for medication, and also prescribing within BNF limits.
2. We however could not evidence proper information sharing with patients (only 40% documented).
3. We could not evidence sufficient information sharing with carers (only 20% documented).
4. PRN medication was mostly prescribed as a range rather than a clear dose, which gave rise to subjective dispensing bias.
5. Side effect monitoring was documented for 85% of patients, meanwhile, the standard for this is 100%.

**Conclusion:**

Clinicians are to ensure that medication information is always shared with service users, and their carers, and this is documented.

Clinicians are to also ensure that PRN medications are prescribed as a single dose rather than as a dose range.

Ward staff are to ensure that they are monitoring side effects and documenting these clearly on electronic notes and ward round documents.

The MDT is to ensure that all regular and PRN medications are reviewed regularly during ward rounds.

Present this audit, share relevant findings with the clinical team, and monitor the implementation of the action plans by doing a reaudit in 6 months.

